# Supplementary material for: A Humanized Yeast Phenomic Model of Deoxycytidine Kinase to Predict Genetic Buffering of Nucleoside Analog Cytotoxicity
Source: Genes (Basel). 2019 Sep 30;10(10):770. doi: 10.3390/genes10100770 (PMC6826991; doi:10.3390/genes10100770)
Supplement: Supplementary file 1 [file genes-10-00770-s001.zip › Additional_Files/Additional_File7_GOTermHeatmaps.zip!/Additional_File7_GOTermHeatmaps/ER_to_Golgi_transport_vesicle.pdf]

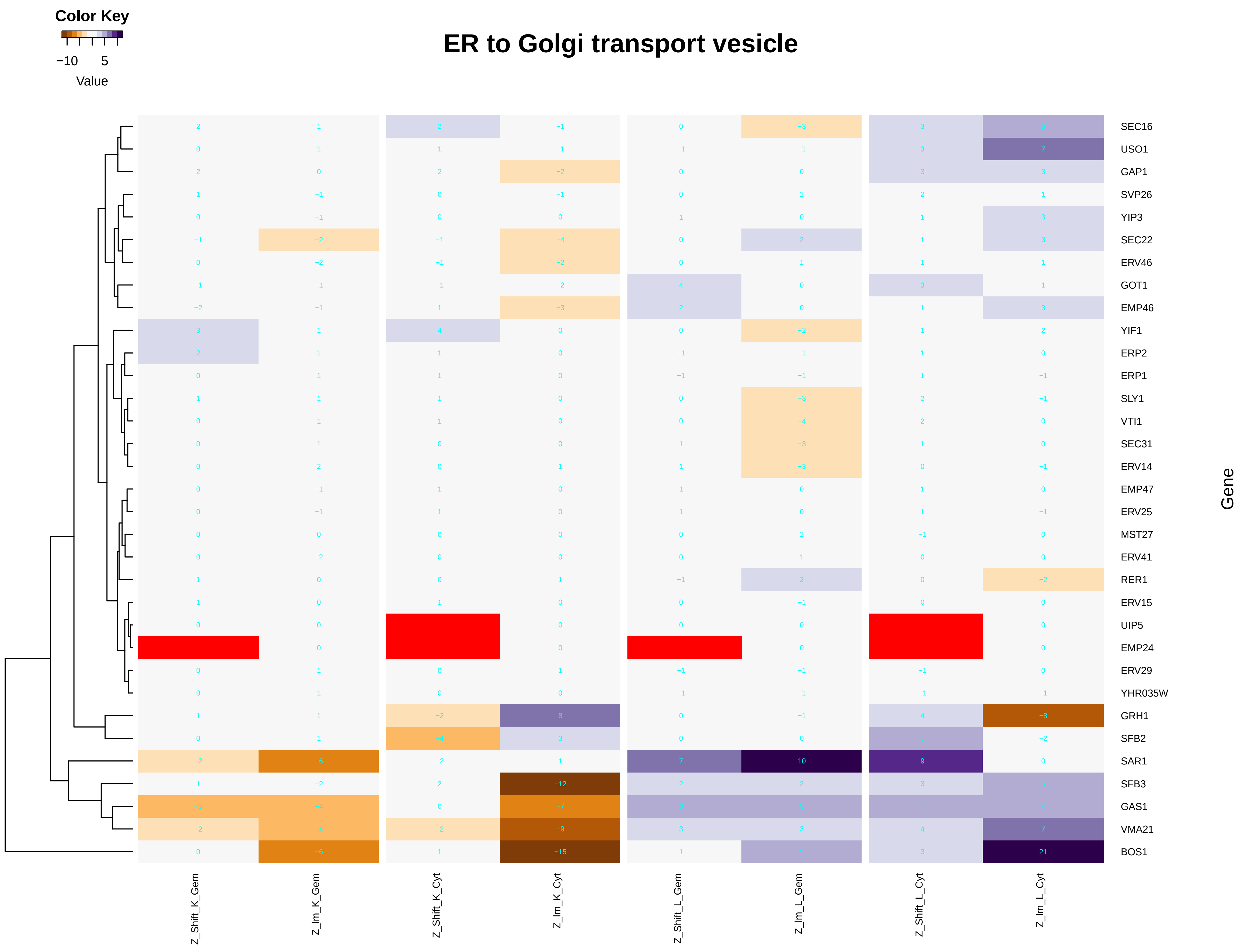
ER to Golgi transport vesicle
Color Key
−10
5
Value
SEC16
USO1
GAP1
SVP26
YIP3
SEC22
ERV46
GOT1
EMP46
YIF1
ERP2
ERP1
SLY1
VTI1
SEC31
ERV14
EMP47
ERV25
MST27
ERV41
RER1
ERV15
UIP5
EMP24
ERV29
YHR035W
GRH1
SFB2
SAR1
SFB3
GAS1
VMA21
BOS1
Gene
Z_Shift_K_Gem
Z_Im_K_Gem
Z_Shift_K_Cyt
Z_Im_K_Cyt
Z_Shift_L_Gem
Z_Im_L_Gem
Z_Shift_L_Cyt
Z_Im_L_Cyt
